# Therapeutic Hypothermia Reduces Oxidative Damage and Alters Antioxidant Defenses after Cardiac Arrest

**DOI:** 10.1155/2017/8704352

**Published:** 2017-05-01

**Authors:** Fernanda S. Hackenhaar, Tássia M. Medeiros, Fernanda M. Heemann, Camile S. Behling, Jordana S. Putti, Camila D. Mahl, Cleber Verona, Ana Carolina A. da Silva, Maria C. Guerra, Carlos A. S. Gonçalves, Vanessa M. Oliveira, Diego F. M. Riveiro, Silvia R. R. Vieira, Mara S. Benfato

**Affiliations:** ^1^Departamento de Biofísica, UFRGS, Porto Alegre, Brazil; ^2^Programa de Pós-Graduação em Biologia Celular e Molecular, UFRGS, Porto Alegre, Brazil; ^3^Grupo Hospitalar Conceição, Porto Alegre, Brazil; ^4^Instituto de Ciências Básicas da Saúde, UFRGS, Porto Alegre, Brazil; ^5^Hospital de Clínicas de Porto Alegre, Porto Alegre, Brazil

## Abstract

After cardiac arrest, organ damage consequent to ischemia-reperfusion has been attributed to oxidative stress. Mild therapeutic hypothermia has been applied to reduce this damage, and it may reduce oxidative damage as well. This study aimed to compare oxidative damage and antioxidant defenses in patients treated with controlled normothermia versus mild therapeutic hypothermia during postcardiac arrest syndrome. The sample consisted of 31 patients under controlled normothermia (36°C) and 11 patients treated with 24 h mild therapeutic hypothermia (33°C), victims of in- or out-of-hospital cardiac arrest. Parameters were assessed at 6, 12, 36, and 72 h after cardiac arrest in the central venous blood samples. Hypothermic and normothermic patients had similar S100B levels, a biomarker of brain injury. Xanthine oxidase activity is similar between hypothermic and normothermic patients; however, it decreases posthypothermia treatment. Xanthine oxidase activity is positively correlated with lactate and S100B and inversely correlated with pH, calcium, and sodium levels. Hypothermia reduces malondialdehyde and protein carbonyl levels, markers of oxidative damage. Concomitantly, hypothermia increases the activity of erythrocyte antioxidant enzymes superoxide dismutase, glutathione peroxidase, and glutathione S-transferase while decreasing the activity of serum paraoxonase-1. These findings suggest that mild therapeutic hypothermia reduces oxidative damage and alters antioxidant defenses in postcardiac arrest patients.

## 1. Introduction

Postcardiac arrest syndrome (PCAS) refers to the pathophysiological consequences of return of spontaneous circulation (ROSC) after successful cardiopulmonary resuscitation (CPR) for cardiac arrest (CA). PCAS comprises brain injury, hemodynamic dysfunction, damage to the heart and other organs, sepsis, and systemic inflammation consequent to ischemia-reperfusion (I/R) injury. The first 3 days after CA are considered the critical phase of PCAS, when patients develop progressive organ damage and increased inflammation [1].

During PCAS, damage to the brain, heart, and other organs resulting from I/R has been attributed to oxidative stress, as the sudden return of oxygen to circulation increases the generation of reactive oxygen species (ROS) [2, 3]. Generation of ROS, especially superoxide radical, increases dramatically after reperfusion and depends on xanthine oxidoreductase (XOR) and other pathways [4, 5]. Endothelial XOR plays a well-known role in free radical generation during hypoxia and reperfusion, when xanthine dehydrogenase (XDH) is converted to xanthine oxidase (XO), using oxygen as an electron acceptor instead of nicotinamide adenine dinucleotide (NAD^+^), thus generating superoxide and hydrogen peroxide [6].

Post-CA hypoxia leads not only to oxidative stress but also to marked metabolic acidosis. Anaerobic glycolysis decreases the glucose concentration within the tissues, while increasing lactate formation. Concomitantly, a compensatory mechanism to restore pH increases the levels of calcium, sodium, and other electrolytes, such as potassium and magnesium [7]. Elevated blood lactate levels are found in patients with poor outcome after CA, since these levels are associated with the time to achieve ROSC [8].

Mild therapeutic hypothermia (MTH) has been the only clinical treatment applied to reduce post-CA injury, and clinical and animal model studies have supported the benefits of MTH for improving brain recovery [9–11]. MTH also prevents damage to other tissues, including the myocardium, liver, and kidney [12]. Damage protection by MTH is reinforced by studies demonstrating reduced oxidative damage after treatment [11–13]. Hypothermia may also be involved in preventing acidosis and lactate clearance, thereby restoring normal metabolism [14, 15].

The present study aimed to investigate oxidative stress during PCAS by assessing oxidative stress parameters at 6, 12, 36, and 72 h after CA in intensive care unit (ICU) patients and by comparing patients treated with controlled normothermia versus MTH.

## 2. Materials and Methods

### 2.1. Screening of Patients

This prospective observational cohort study was conducted between May 2011 and October 2012 and included patients admitted to the ICU of Hospital Conceição, a public hospital located in Porto Alegre, Brazil. The hospital has 843 beds and 59 ICU beds and treats approximately 25,900 patients per year. The study was approved by the institutional ethics committee, and written informed consent was obtained from the relatives of all participants.

The study screened comatose patients, victims of in-hospital or out-of-hospital CA, who had successful ROSC within 20 min of CPR. Patients were assigned to receive either actively controlled normothermia (36°C, *n* = 31) or MTH (33°C, *n* = 11). Patients were assigned to MTH according to the availability of the room equipped for this purpose. All included patients had monitored temperature ≤ 37°C, were in a state of coma (Glasgow Coma Scale score ≤ 8), were receiving adequate oxygenation by mechanical ventilation, as indicated by PaO_2_ ≥ 60 torr (≥8 kPa) with FiO_2_ ≤ 0.4 and positive end-expiratory pressure ≤ 8 cm H_2_O, and were cardiovascularly stable (heart rate ≤ 130 beats/min) with controlled acid-base balance and electrolyte levels. Exclusion criteria were patients aged < 18 years, trauma victims, postoperative patients (<7 days after operation), patients who experienced more than one CA event during admission, pregnant women, and patients with advanced cancer. The full medical history of each patient was evaluated. Long-term survival was followed for 3 years after CA by telephone contact with the patient or family members.

### 2.2. Mild Therapeutic Hypothermia (MTH)

Post-CA MTH was applied according to the hospital protocol. Cooling started 4 h after CA aiming at 32–34°C. A combined cooling method was applied, including the use of endovascular cooling, ice packs, air conditioning, and thermal blanket for controlled cooling and rewarming of patients (circulating-water mattress; Medi-Therm® III Hyper/Hypothermia Machine MTA 6900, Gaymar Industries Inc.). Body temperature was measured continuously by an esophageal temperature probe. Neuromuscular-blocking agents (atracurium and/or pancuronium) were administered to control chills, thereby preventing heating of the body. Rewarming to the target temperature of 36°C started 24 h after hypothermia and did not exceed 0.25–0.5°C/h. Hyperthermia was promptly treated using antipyretic drugs in both normothermic and hypothermic patients, since temperature above 37°C is associated with poor outcome [16].

### 2.3. Clinical and Laboratory Data Assessment and Sample Collection

Clinical and laboratory data and venous blood samples were obtained from both normothermic and hypothermic patients at 6, 12, 36, and 72 h after CA. Clinical data analysis included blood pressure, internal body temperature, heart rate, and respiratory rate. Laboratory data were assessed in venous blood by the hospital biochemistry staff and included pH, glucose, lactate, and electrolyte levels. High-sensitivity C-reactive protein (hs-CRP), hematocrit, and hemoglobin levels were analyzed daily by the hospital biochemistry staff.

Venous blood was drawn from the central venous catheter for analysis of oxidative stress and neuronal injury biomarkers. A total of 10 mL of blood was prepared for isolation of serum, plasma (citrate), and erythrocytes. Samples were stored in liquid nitrogen within 30 min of blood collection and posteriorly stored at −80°C. Erythrocytes were lysed and diluted 1 : 100 in 2% ethanol and 10  *µ*mol/L protease inhibitor phenylmethylsulfonyl fluoride.

### 2.4. Neuronal Injury and Oxidative Stress Biomarkers

#### 2.4.1. Serum S100 Calcium-Binding Protein Beta (S100B) Levels

S100B levels were determined using an in-house ELISA method. Serum samples were coated with anti-S100B monoclonal antibody (SH-B1, Sigma-Aldrich) and then incubated with anti-S100 polyclonal antibody (DAKO), followed by incubation with peroxidase-conjugated anti-rabbit antibody (Sigma-Aldrich). Reaction with o-phenylenediamine was measured using a spectrophotometer at a wavelength of 492 nm [17].

#### 2.4.2. Plasma Carbonyl Levels

Plasma was incubated with 2,4-dinitrophenylhydrazine for analysis of carbonyl content using a spectrophotometer at 370 nm. Hydrazine (ε_370_^M^ 21,000 M^−1^ cm^−1^) was used to determine carbonyl levels [18].

#### 2.4.3. Serum Malondialdehyde (MDA) Levels

Serum MDA levels were measured by high-performance liquid chromatography at 250 nm (LC-18 DB column) [19].

#### 2.4.4. Xanthine Oxidase (XO) Activity

Serum XO activity was measured using a XO fluorometric assay kit (Cayman Chemical, USA), applying an excitation wavelength of 520–550 nm and an emission wavelength of 585–595 nm.

#### 2.4.5. Superoxide Dismutase (SOD) Activity

SOD activity was measured in erythrocyte lysate samples by spectrophotometry using the RanSOD kit (Randox, UK).

#### 2.4.6. Glutathione Peroxidase (GPx) Activity

GPx activity was measured in erythrocyte lysate samples by spectrophotometry using the RanSel kit (Randox, UK).

#### 2.4.7. Glutathione S-Transferase (GST) Activity

GST activity in erythrocyte lysate samples was determined by spectrophotometry by measuring the conjugation of 1-chloro-2,4-dinitrobenzene and glutathione forming S-(2,4-dinitrophenyl)-glutathione (ε_340_^M^ 9600 M^−1^ cm^−1^) [20].

#### 2.4.8. Paraoxonase-1 (PON1) Activity

Serum PON1 activity was measured by spectrophotometry at 412 nm using paraoxon as substrate, based on the formation of p-nitrophenol (ε_412_^M^ 169,000 M^−1^ cm^−1^) by paraoxon hydrolysis [21, 22].

#### 2.4.9. Data Normalization

All assays were performed in triplicate. The results were normalized for total protein content by the Bradford method, using bovine serum albumin as standard [23].

### 2.5. Statistical Analysis

PASW Statistics 18 (SPPS Inc.) was used to construct multivariable logistic regression models with generalized estimating equations in order to compare the parameters of interest. Pairwise comparisons of estimated means by Bonferroni post hoc were used. Two-tailed Spearman's correlation was used to examine the relationship between variables, and the GNU Image Manipulation Program, version 2.8.16, was used to create the schematic representation of the correlations. Variables with normal distribution are presented as mean ± standard error, and variables with nonnormal distribution are presented as median (min; max) or median and interquartile range. The sample size of 42 patients was calculated using the WINPEPI program. COMPARE2 was used to compare the difference of MDA mean ± standard deviation between good and poor outcome groups from a previous study of cardiac arrest patients, to achieve 80% power at a 5% significance level [24].

## 3. Results

### 3.1. Baseline Characteristics and Outcome Biomarkers

Baseline characteristics of patients are shown in Table 1. Age and sex did not differ significantly between normothermic and hypothermic patients. ROSC also did not differ between groups, indicating that participants had similar baseline characteristics in both groups (Table 1).

A total of 42 patients were included in the study. Thirty-one patients received actively controlled normothermia (36°C), while 11 patients received MTH (33°C). In hypothermic patients, body temperature reached 33°C 6 h after CA, and this temperature was maintained for 24 h as observed at 6 and 12 h after CA. After 36 h, body temperature reached 36°C (Table 2).

Blood hs-CRP levels, a biomarker of inflammation, were not significantly altered by hypothermia treatment. Other important biomarkers of outcome after CA were evaluated, including blood levels of calcium, sodium, glucose, and lactate. The levels of lactate, calcium, sodium, and pH were not altered in hypothermic patients. Blood lactate levels decreased significantly 12 h after CA in both groups and remained at the same levels at 36 and 72 h. The decreased blood glucose levels found at the initial 6 and 12 h after CA were significantly increased in patients treated with hypothermia, returning to similar levels at 36 and 72 h after CA in both groups (Table 2).

### 3.2. Oxidative and Brain Damage Biomarkers

**Figure 1:**
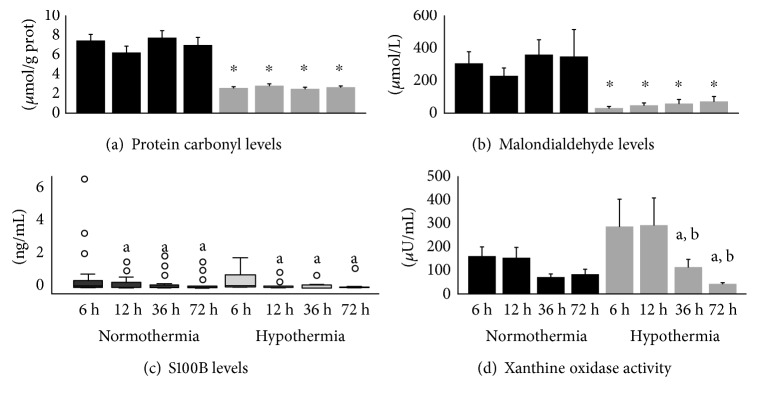
Biomarkers of oxidative damage and brain injury at 6, 12, 36, and 72 h after cardiac arrest (CA) in normothermic (*n* = 31) versus hypothermic (*n* = 11) patients. In hypothermic patients, body temperature reached 33°C at 6 and 12 h and 36°C at 36 and 72 h after CA. (a) Carbonyl levels, a biomarker of oxidative damage to proteins; (b) malondialdehyde levels, a biomarker of oxidative damage to lipids; (c) S100B levels, a biomarker of brain injury; (d) xanthine oxidase (XO) activity, a biomarker of generation of superoxide free radical. Data are expressed as mean ± standard error, except for S100B levels, which are expressed as median and interquartile range. ^○^Significantly different when comparing normothermic versus hypothermic groups at the same time point, *p* ≤ 0.05. ^a^Significantly different within the group when compared to 6 h, *p* < 0.05. ^b^Significantly different within the group when compared to 12 h, *p* ≤ 0.05.

Serum S100B levels, a biomarker of brain injury, did not differ significantly in hypothermic patients. S100B levels decreased significantly 12 h after CA in both groups and remained at similar levels after 36 and 72 h (Figure 1).

MTH clearly reduced the oxidative damage parameters evaluated in the present study. Plasma carbonyl levels, a biomarker of protein oxidative damage, decreased significantly in patients treated with hypothermia at all time points after CA. Likewise, hypothermic patients had decreased serum MDA levels, a biomarker of lipid oxidative damage, at all time points after CA (Figure 1).

XO activity did not differ significantly between hypothermic and normothermic patients. However, in the hypothermic group, XO activity decreased at 36 and 72 h after CA, coinciding with the period of posthypothermia treatment (Figure 1).

#### 3.2.1. Correlation Analysis

**Figure 2:**
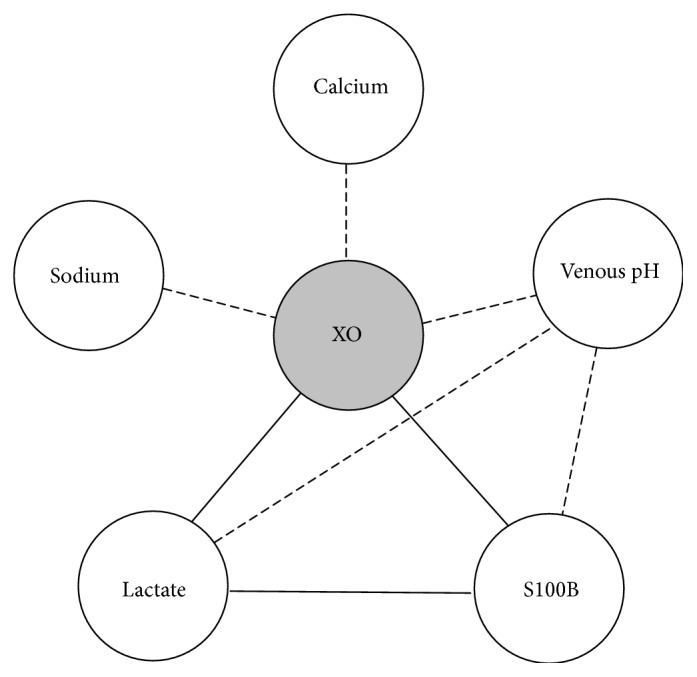
Schematic representation of the correlation analysis of xanthine oxidase (XO) activity. Lines indicate positive (solid lines) and negative (dotted lines) correlations.

Due to its relevance to I/R injury, XO activity was correlated with the biomarkers of outcome and acidosis and the results are summarized in Figure 2. XO activity was negatively correlated with venous pH (*R* = −0.360, *p* ≤ 0.01) and positively correlated with blood lactate (*R* = 0.405, *p* ≤ 0.001); venous pH, in turn, was inversely correlated with blood lactate (*R* = −0.336, *p* ≤ 0.01). Conversely, XO activity was inversely correlated with blood calcium levels (*R* = −0.543, *p* ≤ 0.001) and blood sodium levels (*R* = −0.332, *p* ≤ 0.01). Importantly, serum S100B levels were positively correlated with XO activity (*R* = 0.412, *p* ≤ 0.001) and blood lactate (*R* = 0.408, *p* ≤ 0.001); conversely, serum S100B levels were inversely correlated with venous blood pH (*R* = −0.358, *p* ≤ 0.01). No correlation was found between XO activity and MDA levels (*R* = 0.194, *p* = 0.271) or carbonyl levels (*R* = 0.010, *p* = 0.907).

### 3.3. Antioxidant Enzymatic Activities

**Figure 3:**
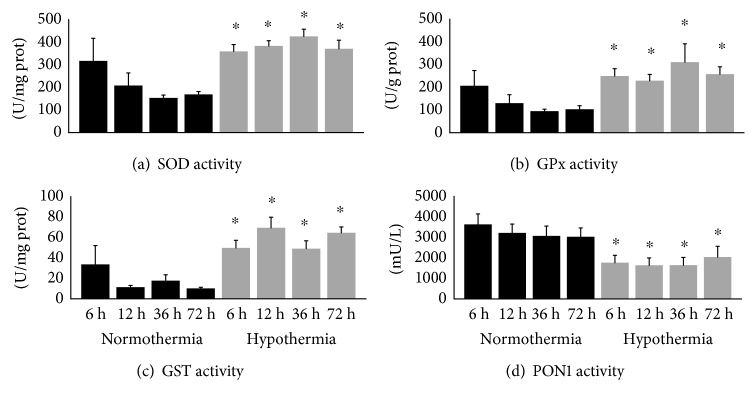
Antioxidant enzymatic activity at 6, 12, 36, and 72 h after cardiac arrest (CA) in normothermic (*n* = 31) versus hypothermic (*n* = 11) patients. In hypothermic patients, body temperature reached 33°C at 6 and 12 h and 36°C at 36 and 72 h after CA. (a) Superoxide dismutase (SOD) activity; (b) glutathione peroxidase (GPx) activity; (c) glutathione S-transferase (GST) activity; (d) paraoxonase-1 (PON1) activity. Data are expressed as mean ± standard error. ^∗^Significantly different when comparing normothermic versus hypothermic groups at the same time point, *p* ≤ 0.05.

The activities of antioxidant enzymes related to the detoxification of ROS were significantly altered by hypothermia treatment. The activities of erythrocyte antioxidant enzymes SOD, GPx, and GST increased significantly at 6, 12, 36, and 72 h after CA in hypothermic patients, as compared to normothermic patients. Surprisingly, PON1 activity, an antioxidant enzyme present in high-density lipoproteins, showed an inverse profile as compared to all other antioxidant enzymes under analysis. Serum PON1 activity was significantly decreased in hypothermic patients at 6, 12, 36, and 72 h after CA (Figure 3).

#### 3.3.1. Correlation Analysis

The activities of erythrocyte antioxidant enzymes were strongly correlated. SOD activity was positively correlated with GPx (*R* = 0.845, *p* ≤ 0.001) and GST (*R* = 0.745, *p* ≤ 0.001) activities. GPx was also strongly correlated with GST activity (*R* = 0.629, *p* ≤ 0.001). Interestingly, serum PON1 activity did not correlate with the activities of the analyzed erythrocyte antioxidant enzymes (SOD, *R* = −0.156, *p* = 0.082; GPx, *R* = −0.097, *p* = 0.288; GST, *R* = −0.154, *p* = 0.085) (data not shown).

## 4. Discussion

The present study evaluated the effects of MTH on oxidative damage and antioxidant profile in post-CA patients. The results suggest that MTH reduces oxidative damage and increases the activity of most of the analyzed antioxidant enzymes. Indeed, carbonyl and MDA levels were significantly reduced while erythrocyte antioxidant enzymes SOD, GPx, and GST showed increased activity during PCAS in the hypothermic group, indicating that hypothermia reduces oxidative stress in post-CA patients.

### 4.1. Oxidative Damage and Xanthine Oxidase (XO) Activity

Oxidative stress has been implicated as a crucial factor in organ damage and hemodynamic dysfunction during PCAS, and, in this setting, MTH may be a suitable treatment option to reduce oxidative stress. MTH was found to decrease frontal cortex MDA and carbonyl levels 24 h after ROSC in a porcine model of CA [11].

In one of the few studies on oxidative stress in post-CA patients treated with hypothermia, plasma reactive oxygen metabolite (d-ROM) levels were decreased during the hypothermic stage of treatment (33°C) [13]. Interestingly, after rewarming, d-ROM levels returned to control-group levels, different from the results reported in the present study, where MDA and carbonyl levels remained lower in the hypothermic group even after rewarming. The difference between these results may indicate that, although high ROS generation returns after rewarming from 24 h MTH, protein damage and lipid peroxidation cascades may be impaired due to the activation of antioxidant defenses by MTH. The d-ROM test indicates the ability of a sample to generate peroxides, called “peroxidizability,” and needs to be carefully analyzed due to possible overestimation of peroxide levels [25]. It is a different methodological approach compared to carbonyl and MDA levels, specific markers of the oxidation of amino acids residues and lipids, respectively.

In a porcine model of CA, MTH also reduced serum reactive oxygen metabolite levels compared to animals under controlled normothermia [12]. In I/R rat models, MDA levels were increased in the liver [26, 27], lung, ileum [27], and skeletal muscle, and these levels were restored by MTH [28]. To the best of our knowledge, no previous study has reported on protein carbonyl as a marker of oxidative damage after CA. Nevertheless, carbonyl levels showed a profile similar to that of MDA levels in the present study, since MTH significantly reduced the levels of both markers during PCAS. These results support the hypothesis that oxidative damage is reduced by MTH.

Despite the well-characterized increase in XO activity generating ROS during I/R injury [29], most studies are still trying to characterize the mechanisms of XO regulation, including the increase in XOR expression [2] and redox modifications due to I/R injury [6, 30]. In this respect, this is a pioneering study evaluating XO activity in post-CA patients who received MTH. No significant difference in XO activity was found during MTH at 6 and 12 h after CA. However, after rewarming (at 36 and 72 h after CA), XO activity was decreased in the hypothermic group. It is important to point out that decreased XO activity at 36 and 72 h in the hypothermic group was not significantly different from that in the normothermic group at the same time points. Further studies are needed to elucidate the effect of temperature on XOR regulation.

Hypoxic acidosis plays a relevant role in XOR regulation [31], but its exact mechanism of action has yet to be described. It is possible that ion imbalance participates in the upregulation of XO in I/R injury together with the changes observed in oxygen saturation. During post-CA acidosis, blood calcium, sodium, and other electrolytes are increased as a compensatory mechanism for high lactate levels [7]. Interestingly, the present results show that XO activity was positively correlated with lactate and inversely correlated with pH, calcium, and sodium, suggesting that XO regulation might have been related to acidosis in our patients. Although XO activity did not correlate with oxidative damage biomarkers in the present study, it was positively correlated with S100B levels. Despite our initial hypothesis that XO activity would correlate with MDA and carbonyl levels, it is possible that the different profiles found in XO activity as compared to the markedly low carbonyl and MDA levels in the hypothermic group may explain this lack of correlation.

### 4.2. Antioxidant Activity

In the present study, the activities of erythrocyte antioxidant enzymes SOD, GPx, and GST showed a similar marked increase after MTH during PCAS, as compared to the normothermic group, and were positively correlated with each other. The activity of antioxidant enzymes is altered as a consequence of I/R injury after CA, and hypothermia may restore impaired defense mechanisms. Despite the paucity of studies evaluating antioxidant defenses in post-CA patients, there is some evidence from I/R models. In rat models of I/R injury, SOD activity was decreased in the liver, lung, and ileum [26–28]. A study in the liver found the same profile for SOD and hydrogen peroxide consumption, but GPx activity increased after I/R injury [26]. Similar to the present results, I/R injury of skeletal muscle in rats decreased the activity of SOD and GPx, and tissue activities were restored by MTH [28]. MTH also increased manganese SOD (MnSOD) expression in the frontal cortex of pigs after CA [11]. No studies were found relating GST, a detoxification enzyme, to CA or I/R injury.

MTH decreased the plasma biological antioxidant potential (BAP) during treatment in post-CA patients, returning to control levels after rewarming [13]. BAP was also evaluated in the serum of animals receiving MTH in a porcine model of CA, but no significant difference was found [12]. The BAP provides a global measurement of many enzymatic and nonenzymatic antioxidants together, indistinctly. Data from both studies do not support our results; however, instead of using the BAP test, we used specific methods for each antioxidant enzyme.

To the best of our knowledge, the present study is the first to evaluate PON1 activity in post-CA patients, and a significant decrease in PON1 activity was observed after MTH. PON1 activity was found to be reduced after I/R injury in the serum, liver, kidney, and lung of rats [32]. In patients undergoing coronary intervention with no-reflow, an I/R event, blood PON1 activity was lower after ischemia than that in patients with normal flow [33]. Interestingly, serum PON1 activity showed an inverse profile as compared to erythrocyte antioxidant enzymes SOD, GPx, and GST. Moreover, there was no correlation between serum PON1 activity and erythrocyte enzymes. These findings suggest that erythrocyte SOD, GPx, and GST are regulated differently from serum PON1 during I/R injury and MTH treatment.

### 4.3. Neuronal Injury and Outcome Biomarkers

Controversy has arisen over the efficacy of MTH following CA. After MTH was recommended by international resuscitation guidelines [34, 35], many studies began to point out that the reduction of neuronal damage by MTH is based on the control of body temperature rather than on hypothermia per se. When comparing treatments at a target temperature of 33°C (473 patients) and 36°C (466 patients), no difference was observed in neurological outcome [36]. However, other studies have pointed out the benefits of MTH by comparing hypothermic to controlled normothermic temperature management. In a porcine model of CA, by comparing controlled temperatures of 33°C and 36.8°C, the 33°C group showed improved brain oxygen saturation and blood pressure, followed by a decrease in organ damage biomarkers in the heart, liver, and kidney. Interestingly, the levels of the marker of neuronal injury, neuron-specific enolase, were not altered by hypothermia [12]. Although S100B is considered a better marker of poor neurological outcome as compared to neuron-specific enolase [37], in the present study, S100B levels were not altered by MTH as well. Control and maintenance of body temperature below 37°C in the normothermic group may explain the similar rates of survival and S100B levels in hypothermic and normothermic patients.

Hs-CRP levels, widely used as marker of inflammation, were not altered by hypothermia. Its levels are inside the range expected for CA patients submitted to MTH [38]; however, no previous studies comparing hs-CRP levels in normothermia versus hypothermia patients were found.

Decreased glucose and elevated lactate levels are well known after CA due to anaerobic glycolysis [7]. Reduced blood glucose levels were found at 6 and 12 h after CA in the normothermic group, and these levels were increased by MTH. Although it is known that hypothermia increases glucose concentration, parsimony is required to evaluate glucose data in post-CA patients, since glucose levels are actively controlled in the ICU.

High lactate levels and impaired lactate clearance are frequently associated with poor outcome after CA [16, 39]. The decreased lactate levels observed after 12 h in the present study were expected due to lactate clearance from the circulation. Lactate levels play an important role in the metabolic acidosis consequent of CA, and the acidosis may be attenuated by increased calcium and sodium levels [7]. In the present study, pH, lactate, calcium, and sodium were not altered by MTH, suggesting hypothermia may not contribute to the attenuation of post-CA acidosis.

### 4.4. Limitations

The main limitation of this study is the lack of a control group of patients who did not undergo CA, making it difficult to discuss the influence of CA alone on oxidative stress parameters. In this respect, experimental models of CA allow a clearer view of PCAS. The study is also limited by the lack of pre-CA biochemical and clinical data, which could not be obtained due to the large number of out-of-hospital CA patients. Another limitation relates to differences in medication, since hypothermic patients received specific medication during MTH. Due to the high complexity of and time required for sample collection, the small number of patients, especially in the hypothermic group, is an important limitation. Finally, the lack of sample collection at a time point closer to the time of CA is also a limitation of the study; however, we determined 6 h after CA as the first sampling time point to avoid disturbing unstable patients and, more importantly, to avoid interrupting patient care.

## 5. Conclusion

In the present study, MTH reduced MDA and protein carbonyl levels as compared to controlled normothermia. An increase in the activity of erythrocyte antioxidant enzymes SOD, GPx, and GST occurred concomitantly with a decrease in the serum antioxidant activity of PON1 in patients treated with MTH. Moreover, hypothermia and acidosis may alter XO activity after CA. Altogether, the present results support our initial hypothesis by providing strong evidence that hypothermia can reduce oxidative stress in post-CA patients.

## Figures and Tables

**Table 1:** Baseline characteristics of 42 comatose in- and out-of-hospital cardiac arrest patients.

	Normothermic (*n* = 31)	Hypothermic (*n* = 11)	All patients (*n* = 42)	*p* value
Age—years (mean ± standard error)	62.6 ± 1.4	60.6 ± 1.6	62.5 ± 1.1	ns
Male patients—*n* (%)	20 (64.5)	7 (63.6)	27 (64.3)	ns
Time to ROSC—min (median ± min; max)	8 ± (2; 20)	6 ± (4; 16)	8 ± (2; 20)	ns
Survival—days (median ± min; max)	8 ± (1; 693)^∗^	16 ± (1; 163)	8 ± (1; 693)	ns
Presumed cause of arrest—number				
Presumed cardiac origin	7/31	3/11	10/42	
Presumed pulmonary origin	6/31	1/11	7/42	
Presumed hemorrhagic origin	1/31	3/11	4/42	
Presumed septic shock origin	5/31	4/11	9/42	
Unspecified origin	12/31		12/42	
Comorbidities—number				
Acquired immunodeficiency syndrome	5/31	1/11	6/42	
Acute biliary pancreatitis	1/31		1/42	
Acute coronary syndrome		1/11	1/42	
Acute renal failure		1/11	1/42	
Acute respiratory infection	2/31		2/42	
Acute respiratory failure		1/11	1/42	
Anemia		1/11	1/42	
Anorexia		1/11	1/42	
Cancer	2/31	4/11	6/42	
Chronic obstructive pulmonary disease	1/31		1/42	
Cirrhosis		1/11	1/42	
Cytomegalovirus	1/31		1/42	
Chronic renal failure	4/31	1/11	5/42	
Congestive heart failure	2/31		2/42	
Diabetes mellitus	2/31	2/11	4/42	
Enterorrhagia	1/31	3/11	4/42	
Gastroenteritis	2/31	2/11	4/42	
Hypertension	3/31		3/42	
Hypothyroidism	1/31		1/42	
Iron deficiency anemia	1/31		1/42	
Ischemic heart disease	3/31	2/11	5/42	
Meningoencephalitis	1/31		1/42	
Myocardial infarction	4/31	1/11	5/42	
Peripheral vascular disease	1/31		1/42	
Pneumonia	2/31	1/11	3/42	
Pulmonary embolism	2/31		2/42	
Sepsis	6/31	4/11	10/42	
Stroke	1/31	1/11	2/42	
Tuberculosis	2/31	2/11	4/42	
Ulcer	1/31	1/11	2/42	

ROSC: return of spontaneous circulation; ^∗^two patients in the normothermic group were alive 3 years after cardiac arrest.

**Table 2:** Biomarkers of outcome in patients with postcardiac arrest syndrome (PCAS).

	Normothermic patients				Hypothermic patients			
	6 h	12 h	36 h	72 h	6 h	12 h	36 h	72 h
Temperature, °C	35.6 ± 0.27	35.7 ± 0.21	35.6 ± 0.53	36.1 ± 0.15	32.9 ± 0.25^**+,#**^	32.6 ± 0.31^**+,#**^	36.1 ± 0.30	36.1 ± 0.20
Hs-CRP, mg/L	114.6 ± 20.92	n.a.	178.4 ± 36.1	141.2 ± 44.3	173.4 ± 31.7	n.a.	161.8 ± 38.82	205.9 ± 65.18
S100B, ng/mL	0.13 (0.01;6.63)^**+**^	0.07 (0.01;1.42)	0.06 (0.001;1.8)	0.50 (0.01;1.3)	0.15 (0.02;1.8)^**+**^	0.09 (0.04;0.71)	0.04 (0.01;0.6)	0.03 (0.02;1.10)
Venous pH	7.28 ± 0.04	7.36 ± 0.04	7.33 ± 0.06	7.35 ± 0.03	7.20 ± 0.05	7.28 ± 0.05	7.34 ± 0.043	7.32 ± 0.07
Glucose, mg/dL	123.6 ± 16.03^+^	126.6 ± 8.47^+^	138.9 ± 9.35	167.3 ± 24.11	179.2 ± 22.43^**+,#**^	155.3 ± 22.43^**#**^	129.0 ± 8.42	107.3 ± 11.22
Lactate, mmol/L	2.9 (0.8;13.7)^**+**^	1.8 (0.7;17)	1.4 (0.7;11.8)	1.7 (0.5;8.5)	1.9 (0.6;18)^**+**^	1.8 (0.6;19)	1.6 (1.0;4.2)	1.4 (0.6;1.5)
Sodium, mmol/L	136.4 ± 1.54	136.9 ± 1.48	136.2 ± 1.70	134.0 ± 2.11	135.5 ± 2.46	104.1 ± 1.98	134.6 ± 3.36	140.3 ± 1.70
Calcium, mg/dL	1.04 ± 0.025	1.09 ± 0.07	1.05 ± 0.04	1.06 ± 0.06	1.05 ± 0.02	1.04 ± 0.02	1.03 ± 0.03	1.13 ± 0.03

Hs-PCR: high-sensitivity C-reactive protein; data are expressed as mean ± standard error, except for S100B and lactate, which are expressed as median (min; max); + is significantly different among time points within the normothermic or hypothermic group, *p* ≤ 0.05; # is significantly different when comparing normothermic versus hypothermic groups at the same time point, *p* ≤ 0.05.

## Abbreviations

BAP: Biological antioxidant potential
CA: Cardiac arrest
CPR: Cardiopulmonary resuscitation
GPx: Glutathione peroxidase
GST: Glutathione S-transferase
hs-CRP: High-sensitivity C-reactive protein
ICU: Intensive care unit
I/R: Ischemia-reperfusion
MDA: Malondialdehyde
MTH: Mild therapeutic hypothermia
NAD^+^: Nicotinamide adenine dinucleotide
PCAS: Postcardiac arrest syndrome
PON1: Paraoxonase-1
ROS: Reactive oxygen species
ROSC: Return of spontaneous circulation
S100B: S100 calcium-binding protein beta
SOD: Superoxide dismutase
XO: Xanthine oxidase
XOR: Xanthine oxidoreductase.
